# Phenotypic and Genotypic Characterization of Resistance and Virulence Markers in *Candida* spp. Isolated from Community-Acquired Infections in Bucharest, and the Impact of AgNPs on the Highly Resistant Isolates

**DOI:** 10.3390/jof10080563

**Published:** 2024-08-09

**Authors:** Viorica Maria Corbu, Ana-Maria Georgescu, Ioana Cristina Marinas, Radu Pericleanu, Denisa Vasilica Mogos, Andreea Ștefania Dumbravă, Liliana Marinescu, Ionut Pecete, Tatiana Vassu-Dimov, Ilda Czobor Barbu, Ortansa Csutak, Denisa Ficai, Irina Gheorghe-Barbu

**Affiliations:** 1 Faculty of Biology, University of Bucharest, Intrarea Portocalelor No. 1-3, 060101 Bucharest, Romania; viorica-maria.corbu@bio.unibuc.ro (V.M.C.); pericleanuradu@gmail.com (R.P.); d.mogos20@s.bio.unibuc.ro (D.V.M.); andreeadum29@gmail.com (A.Ș.D.); tatiana.vassu@bio.unibuc.ro (T.V.-D.); ilda.barbu@bio.unibuc.ro (I.C.B.); ortansa.csutak@bio.unibuc.ro (O.C.); irina.gheorghe@bio.unibuc.ro (I.G.-B.); 2 The Research Institute of the University of Bucharest (ICUB), 050095 Bucharest, Romania; ioana.cristina.marinas@gmail.com; 3 Faculty of Chemical Engineering and Biotechnologies, National University of Science and Technology Politechnica of Bucharest, 060042 Bucharest, Romania; liliana10marinescu@gmail.com (L.M.); denisaficai@yahoo.ro (D.F.); 4 Central Reference Synevo-Medicover Laboratory, 021408 Bucharest, Romania; pecete.ionut@yahoo.ro; 5 Academy of Romanian Scientists, 3 Ilfov Street, 050045 Bucharest, Romania

**Keywords:** community-acquired infections, dermatomycosis, *Candida albicans*, *Candida* non-*albicans*, virulence markers, antifungals, silver nanoparticles

## Abstract

Background: This study aimed to determine, at the phenotypic and molecular levels, resistance and virulence markers in *Candida* spp. isolated from community-acquired infections in Bucharest outpatients during 2021, and to demonstrate the efficiency of alternative solutions against them based on silver nanoparticles (AgNPs). Methods: A total of 62 *Candida* spp. strains were isolated from dermatomycoses and identified using chromogenic culture media and MALDI-TOF MS, and then investigated for their antimicrobial resistance and virulence markers (VMs), as well as for metabolic enzymes using enzymatic tests for the expression of soluble virulence factors, their biofilm formation and adherence capacity on HeLa cells, and PCR assays for the detection of virulence markers and the antimicrobial activity of alternative solutions based on AgNPs. Results: Of the total of 62 strains, 45.16% were *Candida parapsilosis*; 29.03% *Candida albicans*; 9.67% *Candida guilliermondii*; 3.22% *Candida lusitaniae*, *Candia pararugosa,* and *Candida tropicalis*; and 1.66% *Candida kefyr*, *Candida famata*, *Candida haemulonii*, and *Candida metapsilosis*. Aesculin hydrolysis, caseinase, and amylase production were detected in the analyzed strains. The strains exhibited different indices of adherence to HeLa cells and were positive in decreasing frequency order for the *LIP1*, *HWP1*, and *ALS1*,*3* genes (*C. tropicalis*/*C. albicans*). An inhibitory effect on microbial growth, adherence capacity, and on the production of virulence factors was obtained using AgNPs. Conclusions: The obtained results in *C. albicans* and *Candida* non-*albicans* circulating in Bucharest outpatients were characterized by moderate-to-high potential to produce VMs, necessitating epidemiological surveillance measures to minimize the chances of severe invasive infections.

## 1. Introduction

Around 150 species belong to the genus *Candida*, with only a few regarded as human pathogens, and with most being part of the normal human microbiome, present on the oral mucosa as well as in the gastrointestinal, urinary, and genital systems. However, host defense alterations can lead to superficial infections in immunocompetent individuals or systemic infections in immunocompromised patients [1,2]. Candidemia is the fourth most common cause of hospital-acquired bloodstream infections in the United States [1,3] and ranks seventh in Europe, with mortality rates between 22 and 75% [1]. *Candida* spp. cause a wide range of infections globally, from superficial to severe systemic illnesses [4]. In Eastern Europe, the incidence of candidiasis is concerning due to population density, healthcare infrastructure, and antimicrobial usage. Understanding the characteristics of *Candida* isolates from community-acquired infections is crucial for assessing antifungal susceptibility and informing treatment strategies. Furthermore, in Bucharest (the capital city of Romania), limited information exists on the characterization of *Candida* isolates from community-acquired infections such as dermatomycoses. 

Recently, in Romania, high levels of antibiotic resistance have been reported in ESKAPE pathogens (*Enterococcus faecium*, *Staphylococcus aureus*, *Klebsiella pneumoniae*, *Acinetobacter baumannii*, *Pseudomonas aeruginosa*, *Enterobacter* spp.), indicating that there is a major risk for the Romanian population to also be affected by fungal infections. Normally, antibiotics are used to treat bacterial infections by killing or inhibiting the growth of bacteria. However, their use can inadvertently impact fungal infections, particularly by disrupting the natural balance of microorganisms in the body. Antibiotics can eliminate beneficial bacteria that compete with fungi like *Candida*, creating an environment where these fungi can thrive and proliferate. This imbalance can lead to an overgrowth of fungi, resulting in infections such as candidiasis. Therefore, while antibiotics are essential for combating bacterial diseases, their usage can inadvertently contribute to the proliferation of fungal infections by disturbing the microbial ecosystem [5,6].

Despite *C. albicans* being the primary species responsible for infections [7], there has been an increase in *Candida* non-*albicans* (NAC) species such as *Candida glabrata*, *Candida tropicalis*, *Candida krusei*, *Candida parapsilosis*, *Candida kefyr*, and *Candida dubliniensis* [1,7,8]. The pathogenicity of *Candida* spp. involves virulence traits like adherence, biofilm formation, secretion of hydrolytic enzymes, yeast-to-hyphae transition, and immune evasion [9]. Adherence to host cells is a critical first step in the development of infection, with *Candida* spp. also adhering to medical device surfaces, leading to biofilm formation [7,10]. Cell wall proteins like adhesins (*ALS* and *EPA*) and hyphal wall protein (*HWP1*) play crucial roles in adherence and biofilm development [7].

Hydrolytic enzymes are linked to adherence and host cell damage [11]. Secreted aspartyl proteases (SAPs) degrade multiple human proteins, while phospholipases hydrolyze cell membrane phospholipids, leading to tissue invasion. Hemolysins facilitate iron acquisition, vital for fungal survival [10]. Lipases, particularly *LIP1* and *LIP4*, contribute to virulence through host cell adherence, tissue invasion, and lipid utilization [12,13,14].

Antifungal resistance mechanisms in *Candida* spp. include modifications of cell wall components and efflux pump overexpression. Echinocandins target cell wall synthesis, but *FKS* gene mutations confer resistance [15,16]. Azole resistance involves multiple mechanisms, such as efflux pump overexpression and enzyme mutations [15]. Antifungal resistance is a global issue, necessitating surveillance to guide therapy decisions [17,18]. *Candida* biofilms contribute to resistance and immune evasion, emphasizing the need for genotypic characterization to understand resistance and virulence mechanisms [19,20,21]. 

Combining phenotypic and molecular methods provides a robust framework for understanding antifungal resistance and virulence in *Candida* spp. isolates.

Phenotypic methods (chromogenic culture media; disk diffusion; epsilometer test; broth microdilution; VITEK 2; biofilm and hyphal formation assays) are essential for determining the resistance and virulence profiles and guiding clinical treatment decisions [22,23,24,25,26,27,28,29,30,31,32]. Molecular methods provide precise and detailed insights into the genetic and functional mechanisms underlying antimicrobial resistance and virulence in *Candida* spp. isolates and are based on the following: conventional PCR; real-time PCR; Sanger sequencing, used to sequence specific genes that confer resistance, such as *ERG11*, *FKS1*, and *FKS2*, which are associated with azole and echinocandin resistance, respectively; next-generation sequencing, which provides comprehensive analysis of the entire genome; proteomics (MALDI-TOF mass spectrometry for the identification of fungal species and resistance profiles based on protein spectra); molecular typing methods based on multilocus sequence typing, used for epidemiological studies; and whole-genome sequencing, which provides detailed genetic information about the pathogen, allowing for the detection of all possible resistance genes and mutations, particularly useful for epidemiological studies and understanding the genetic basis of resistance; genetic manipulation techniques such as CRISPR-Cas9, used to create gene knockouts to study the function of virulence genes; or RNA interference (RNAi), which silences specific genes to observe changes in virulence traits [33,34,35]. Integrating phenotypic and molecular methods provides a comprehensive understanding of fungal pathogens’ resistance and virulence, which is crucial for developing effective treatments and improving the diagnosis, treatment, and control of infections.

While there are multiple strategies to control *Candida* infections, their effectiveness can be compromised by factors related to the pathogen (e.g., exhibiting different virulence and susceptibility levels), the host (e.g., the individual’s immune status, comorbidities, and overall health), the environment (humidity and warmth), and patient compliance with prescribed treatments and lifestyle modifications. Ongoing research and individualized treatment plans are essential to improve outcomes in managing *Candida* infections [36,37]. One possible strategy is represented by metallic nanoparticles, among which silver nanoparticles (AgNPs) are effective against *Candida*, interfering with virulence factors and biofilm formation [38,39,40,41]. AgNPs inhibit extracellular enzyme production and hemolysin activity, which are crucial for pathogenesis [42,43]. They also disrupt biofilms, enhancing susceptibility to antifungal treatment [44,45].

This study aims to investigate resistance and virulence markers in *C. albicans* and NAC provided by the Central Reference Synevo-Medicover, and to evaluate the effectiveness of AgNPs as an alternative treatment.

## 2. Materials and Methods

### 2.1. Strains Identification and Analysis

**Figure 1:**
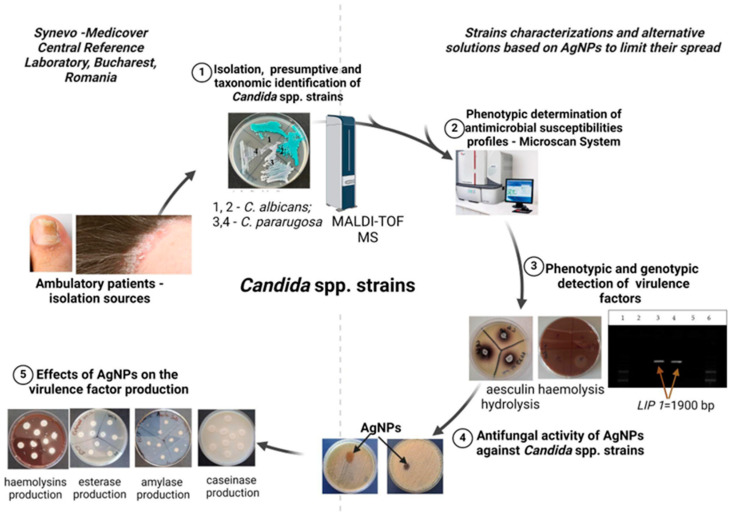
The experimental design (created with Biorender.com; accessed on 11 June 2024).

A total of 62 *Candida* spp. strains were provided by the Central Reference Synevo-Medicover (Bucharest, Romania) and previously isolated from cutaneous infections represented by nail fragments, interdigital scales, and scales from ambulatory patients during 2021 (Supplementary Table S1) The isolates’ identification was performed based on presumptive characteristics on chromogenic culture media using CHROMagarTM *Candida* (CHROMagar, Paris, France), and taxonomic identification was carried out using MALDI-TOF MS MBT Smart, with MSP 96 target polished steel BC (Bruker system, Berlin, Germany) (see Figure 1). 

The investigated *Candida* spp. strains were maintained in a Revco LegaciTM Refrigeration System (Copeland, UK) at −80 °C on Sabouraud (Sab, Merck, Darmstadt, Germany) medium supplemented with 20% glycerol. Before their use, the *Candida* strains were cultivated for 24 h at 37 °C on Sab medium to gain insights into their behavior, survival mechanisms, and pathogenic potential in the human body.

### 2.2. Antibiotic Susceptibility Testing

The antifungal resistance patterns of *Candida* spp. strains were evaluated using Integral System Yeast Plus (Liofilchem, Roseto degli Abruzzi, Italy). This system assesses the growth inhibition of strains in media containing different antifungals and a growth indicator. Susceptibility profiles were determined by observing the color changes after inoculating a standardized suspension (1 McFarland) from the tested strains into sterile water and incubating at 37 °C for 48 h. The color indicators were red for sensitive, orange for intermediate, and yellow for resistant. The antifungals tested, and their specific concentrations, were as follows: nystatin (1.25 µg/mL), amphotericin B (2 µg/mL), flucytosine (16 µg/mL), econazole (2 µg/mL), ketoconazole (0.5 µg/mL), clotrimazole (1 µg/mL), miconazole (2 µg/mL), itraconazole (1 µg/mL), voriconazole (2 µg/mL), and fluconazole (64 µg/mL).

### 2.3. Soluble Virulence Factors: Enzyme and Organic Acid Production

The soluble virulence factors, i.e., metabolic enzymes (amylase, lipase, gelatinase, hemolysins, aesculin hydrolysis, caseinases, and lecithinase) in *Candida* spp. were assessed on specific solid culture media as previously described, on Sab medium supplemented with 5% sheep blood (hemolysin production revealed by the colorless area around the culture after incubation for 24 h at 37 °C), 2.5% yolk agar (lecithinase activity: a clear zone around the inoculated culture after incubation for 24 h at 37 °C), 1% Tween 80 (Sigma-Aldrich, Burlington, MA, USA; lipase production: a precipitation zone around the spot after incubation for 24 h at 37 °C), 15% soluble casein (caseinase activity: a white precipitate surrounding the culture after incubation for 24 h at 37 °C), 3% gelatine (Merck, Germany; gelatin hydrolysis: a colorless zone around the culture after incubation for 24 h at 37 °C), 1% starch (amylase activity: yellow ring around the culture, while the rest of the plate will be blue after adding Lugol’s solution; Sigma-Aldrich, USA), and Fe^3+^ citrate (aesculin hydrolysis: a black precipitate around the culture after incubation for 24 h at 37 °C) [46,47,48].

### 2.4. Biofilm Formation Capacity

The quantification of biofilm formation capacity was assessed as described by Stepanović et al. For this, 20 μL of a 1-McFarland suspension from 24 h *Candida* spp. cultures grown at 37 °C on Sab medium was inoculated onto Roswell Park Memorial Institute 1640 Medium (RPMI 1640) to a final volume of 120 μL in 96-well microtiter plates. After incubating for 24 h at 37 °C, the plates were washed three times with phosphate-buffered saline (PBS). To facilitate fixation, the cells were treated with methanol for 5 min. Staining was then conducted using a 1% solution of crystal violet (Sigma, USA) for 15 min. Following three additional PBS washes, the cells were resuspended in 33% acetic acid solution. Absorbance (OD) values were measured at 490 nm using the Thermo Scientific Multiskan FC spectrophotometer. All samples were analyzed in triplicate, and the relative biofilm-forming capacity of each strain was assessed as previously described in [49]. The reference strains of *C. albicans* (ATCC 10231) and *C. parapsilosis* (ATCC 22019) purchased from the American Type Culture Collection (ATCC, Washington, DC, USA) were used as positive controls for biofilm producers. 

### 2.5. Investigation of Adherence Capacity on HeLa Cells 

A modified Cravioto method was used to assess the adherence capacity of the selected *Candida* spp. strains to HeLa cells (ATCC CCL-2, USA), as previously described [50]. HeLa cells were cultured in 6-well plates with Dulbecco’s Modified Eagle’s Medium (Sigma-Aldrich, USA) and antibiotics for 24 h until 80% confluence. After washing the wells thrice with PBS, we prepared the fungal suspension by incubating yeast in SAB glucose broth at 37 °C for 48 h. The yeast cells were centrifuged, washed, and resuspended to a density of 10^7^ CFU/mL. We then added 1 mL of this suspension to each well and incubated the plates at 37 °C for 2 h to facilitate yeast adherence. Post-adherence, the cells were fixed with methanol and stained with Giemsa solution (Sigma, Darmstadt, Germany). After drying, the samples were examined under a microscope, using a wet objective (×1000 magnification), and photographed. The adherence patterns to eukaryotic cells were defined as follows: localized adherence, characterized by clusters of *Candida* cells to specific areas on the surface of HeLa cells; aggregative adherence, characterized by microorganisms’ adhesion to the HeLa cell surface in overlapping structures; and diffuse adherence, characterized by a widespread attachment of *Candida* cells, covering the entire surface of the HeLa cell. The adherence index (AIn) and the average number of fungal cells per HeLa cell were calculated.

### 2.6. Genotypic Investigation of Virulence Markers Involved in Adherence, Lipolytic Activity, or Genes Encoding β-1,3-glucan Synthase

Simplex and multiplex PCR were used to identify genes associated with adherence (*ALS1*, *ALS3*, *HWP1*) and lipolytic activity (*LIP1*, *LIP4*). DNA templates were extracted using a protocol developed by Csutak et al. (2014) [51] and amplified using specific primers and programs outlined in Supplementary Tables S2 and S3. The presence of these genes was verified through gel electrophoresis. Positive strains were used as positive controls (*C. albicans* ATCC 10231 for ALS1 and 3, and *C. tropicalis* CMGB165 for *LIP1* and 4).

### 2.7. Evaluation of Antimicrobial and Anti-Biofilm Activity, and the Influence of AgNP Solutions (AgNPs) on Metabolic Activity 

#### 2.7.1. Synthesis and Characterization of AgNPs

Approximately 0.888 g of PVP K 30 was dissolved in 80 mL of PEG 400, agitated, and heated to 80 °C until the solution turned clear. At this temperature, 2 mL of 0.5 M AgNO_3_ was quickly added, producing a dark yellow hue. Stirring continued at 80 °C until the mixture had turned dark brown. The solution was then transferred to a Teflon vat and heated to 220 °C under 1 bar pressure for 2 h. After cooling, the reddish-brown liquid was removed from the vat, yielding 1 mg/mL nanoparticle solution. The silver nitrate (AgNO_3_), sodium borohydride (NaBH_4_), polyvinylpyrrolidone (PVP), and polyethylene glycol (PEG 400) were acquired from Roth, hydrogen peroxide (30% H_2_O_2_) from Silal Trading, trisodium citrate (Na_3_C_6_H_5_O_7_) from Alfa Aesar, and 1-butanol from Merck [38]. All reagents and solvents were used without further purification.

For the characterization of AgNPs, FTIR, SEM, TEM, DLS, and XRD methods were used as described by Corbu et al. in 2023 [52].

Before any experiments involving the AgNPs solution, an ultrasonic bath (Daihan Scientific, Seoul, Republic of Korea) set to a frequency range between 20 and 40 kHz for 10 min was used in order to obtain an homogeneous dispersion.

#### 2.7.2. Qualitative Antimicrobial Activity

A modified diffusion method was conducted to perform a rapid screening regarding the antimicrobial activity of AgNPs against a total of 62 strains of *Candida* spp. From 24 h cultures 1-McFarland microbial suspensions were prepared in sterile distilled water, and then each suspension was evenly spread on Sab medium. Subsequently, 10 μL of 1 mg/mL AgNPs was spotted over. The plates were incubated at 37 °C for 24 h, and at the end of the incubation time the diameters of the growth inhibition zones were measured and converted into arbitrary units: 0 for no inhibition, 1 for a growth inhibition zone diameter up to 10 mm, and 2 for a growth inhibition zone diameter of 11–20 mm [50,53,54].

#### 2.7.3. Quantitative Antimicrobial Activity

A total of 9 strains selected as being sensitive to AgNPs and as representative for each *Candida* species were used in order to determine the minimal inhibitory concentration of AgNPs. The quantitative antimicrobial activity was assessed in RPMI 1640 broth medium (American Biorganics, Buffalo, NY, USA) by performing serial twofold microdilutions of AgNPs in 100 μL of the medium (ranging between 500 and 0.97 µg/mL). This broth was inoculated in the next step with 20 μL of a 1-McFarland suspension from 24 h cultures grown at 37 °C on Sab medium. The growth was monitored using a BioTek Synergy HTX multi-reader (Santa Clara, CA, USA) by determining the optical density at 600 nm. The positive (untreated cultures) and negative controls (sterility control) were included, and the minimum inhibitory concentration (MIC) values were determined after incubation for 24 h at 37 °C as the last concentration for which no growth was recorded.

#### 2.7.4. The Influence of AgNPs on Adherence Capacity 

*Candida* spp. strains treated with sub-inhibitory concentrations of AgNPs (MIC/2 and MIC/4) were assessed for their ability to produce soluble virulence factors (hemolysins, amylase, lipase, caseinase, and aesculin hydrolysis) using 10 μL of 1-McFarland suspensions from strains cultured for 24 h at 37 °C on Sab medium [untreated cultures (serving as growth controls) and cultures treated with AgNPs]. After incubating for 24 h at 37 °C, the influence on virulence factor production was determined, based on previously described relations [46,55]. For the determination of the percentage inhibition of *Candida* adherence (PICA%), we used the same protocol as for the biofilm formation capacity (Section 2.4) and the following relationship: PICA% = (As-Ablank)*100/(Ac-Ablank), where As is the absorbance value of samples treated with sub-inhibitory concentrations of materials at 490 nm, and Ac is the absorbance value of the control (microbial strain not treated with materials) at 490 nm, as previously revealed in [46,55].

#### 2.7.5. The Influence of AgNPs on the Viability of Microorganisms in Biofilms

To assess the viability of microbial cells in biofilms, a colorimetric method based on the reduction of a tetrazolium salt was used, as described in [56]. After incubation in microtiter plates, the microbial suspension was removed, and 150 μL of PBS and 50 μL of MTT 0.3% were added for 2 h at 37 °C. After removing the MTT solution, 150 μL of DMSO and 25 μL of 0.1 M glycine buffer (pH 10.2) were added. The absorbance was measured at 550 nm. For the determination of the percentage inhibition of *Candida* viability (PICV%), the equation used was PICV% = (As-Ablank)*100/(Ac-Ablank), where As is the absorbance value of samples treated with sub-inhibitory concentrations of materials, and Ac is the absorbance value of the control (microbial strain not treated with materials).

### 2.8. Extracellular Nitric Oxide Quantification

The extracellular nitric oxide (NO) was measured using previously published techniques, with certain modifications [57]. The NO release was determined by a spectrophotometric assay using the Griess reagent. After the incubation period, the microbial suspension was centrifuged at 6739 g for 10 min. The supernatant was mixed with 50 μL of 2% sulfanilamide (Sigma-Aldrich, Germany) in 5% (*v*/*v*) H_3_PO_4_ and 50 μL of 0.1% N-(1-naphthyl)-ethylenediamine aqueous solution (Sigma-Aldrich, Germany). After 30 min, the azo dye was measured at λ = 540 nm. To quantify the nitric oxide, a calibration curve was developed using NaNO_2_ (Sigma-Aldrich Chemie GmbH, Darmstadt, Germany) at concentrations ranging from 1 to 100 μM (R2 = 0.9971).

### 2.9. Statistical Analysis

Data collected and analyzed in triplicate were presented as means ± SD, and statistical analysis was conducted using GraphPad Prism v10 (GraphPad Software, San Diego, CA, USA). For assessing adherence and virulence factor inhibition, a two-way ANOVA was applied. Dunnett’s multiple comparisons test was used to adjust for multiple comparisons in evaluating the effects of AgNPs at sub-inhibitory concentrations on *Candida* adherence to inert substrata and the inhibition of virulence factor production, comparing control values with MIC/2 and MIC/4 values for each isolation source. Additionally, extracellular nitric oxide content was analyzed using two-way ANOVA, with Tukey’s method for multiple comparisons corrections using a single pooled variance, comparing samples to strain controls. Correlations among extracellular nitric oxide content, adherence, and virulence factor inhibition were determined using Pearson correlation. The threshold for statistical significance was established at *p* < 0.05.

## 3. Results

### 3.1. Phenotypic and Genotypic Features of Resistance and Virulence Markers in Candida spp. Isolates

In this study, 62 strains of *Candida* spp. were previously isolated from dermatomycoses and identified using chromogenic CHROMagarTM Candida media and by mass spectrophotometry (MALDI-TOF MS), in the following decreasing frequency order: 45.16% *C. parapsilosis*; 29.03% *C. albicans*; 9.67% *C. guilliermondii*; 3.22% *C. pararugosa*, *C. tropicalis*, and *Candida lusitaniae*; 1.66% *Candida haemulonii*, *Candida famata*, *Candida metapsilosis*, and *Candida kefyr*.

The analyzed strains were isolated mostly from women (62.90% of cases) aged between 15 and 77 years. Also, the main source of isolation from the ambulatory patients treated in Bucharest was the right hand nail (40.32%), followed by the right foot (22.58%), left foot (16.12%), left hand nail (14.51%), and scalp and face (3.22%) (see Table 1).

The antifungal susceptibility profiles determined using Integral System Yeast Plus demonstrated the following patterns, by species: *C. parapsilosis* isolates were resistant to amphotericin B (29%), clotrimazole (24%), nystatin (18%), econazole (11%), miconazole (7%), and itraconazole (4%); *C. albicans* to amphotericin B (22%), clotrimazole (27%), nystatin (17%), econazole (11%), and miconazole (6%); *C. tropicalis* to clotrimazole, econazole, and nystatin (50/50/%); *C. guilliermondii* to nystatin (17%); *C. lusitaniae* to nystatin and amphotericin B (100%); and *C. haemulonii* to clotrimazole and econazole (100%) (see Table 1). At the opposite side, *C. parapsilosis* strains were sensitive only to ketoconazole, voriconazole, and fluconazole; *C. albicans* to flucytosine, ketoconazole, itraconazole, voriconazole, and fluconazole; *C. tropicalis* to amphotericin B, flucytosine, ketoconazole, miconazole, itraconazole, voriconazole, and fluconazole; *C. guilliermondii* to all antifungals except nystatin; *C. krusei* to all antifungals tested; along *C. metapsilosis*, *C. pararugosa, C. famata*, and *C. haemulonii* to all except econazole and clotrimazole; and *C. lusitaniae* to all antifungals except amphotericin B and nystatin.

Virulence factors tested on specific culture media supplemented with various nutrients revealed that *Candida* spp. strains were positive for aesculin hydrolysis (93.35%), caseinase (79.03%), amylase (35.48%), hemolysins (30.65%), and lipase production (20.79%). The virulence factors’ distribution by species and isolation sources was as follows: *C. albicans*, isolated from feet, hand nails, and face scales was positive for aesculin hydrolysis, hemolysins, amylase, and lipase; *C parapsilosis* from feet, left hand nails, and scalps was positive for aesculin hydrolysis, caseinase, hemolysins, amylase, and lipase; *C. tropicalis*, isolated from left feet, was positive for aesculin hydrolysis, caseinase, hemolysins, and amylase; *C. guilliermondii* from left feet, hand nails, and scalps was positive for aesculin hydrolysis, caseinase, hemolysins, amylase, and lipase; *C. lusitaniae* from right hand nails was positive for aesculin hydrolysis, caseinase hemolysins, amylase, and lipase; *C. krusei* from left hand nails was positive for aesculin hydrolysis and amylase; *C. metapsilosis* from right hand nails was positive for aesculin hydrolysis and caseinase; *C. famata* from left feet was positive for caseinase; and *C. pararugosa* isolated from left and right hand nails was positive for aesculin hydrolysis (see Table 2).

Based on their biofilm formation capacities, the studied strains of *C. albicans* and *NAC* were classified into the following categories: 20.64% did not produce biofilms (NP), while 37.09% were categorized as weak biofilm producers (W), 1.61% as moderate biofilm producers (M), and 30.64% as strong biofilm producers (Table 3). 

The *Candida* spp. strains included in our study were also investigated for their adherence patterns to eukaryotic cells (HeLa cells). It was found that most of the strains presented all investigated adherence patterns, in the following decreasing order: diffuse (47.8%), localized (29.7%), and aggregative (22.5%). The adherence index (AIn) ranged from 26% to 50%, indicating that the data correspond to the high frequency of diffuse adherence pattern identification (see Table 3).

Genotypic assays were performed for the targeted identification of virulence markers such as the *ALS1*, *ALS3*, *HWP1*, *LIP1*, and *LIP4* genes. The results showed that the strains were positive i for the following genes, in decreasing order of frequency: *LIP1* (100% of *C. tropicalis*), *HWP1*, and *ALS3* (82.35% of *C. albicans*) (see Table 2).

### 3.2. Antimicrobial and Anti-Biofilm Activity, and the Impact of AgNP Solutions on the Adherence Capacity and Metabolic Activity

#### 3.2.1. Qualitative and Quantitative Antimicrobial Activity of AgNPs against *Candida* spp. Strains

**Figure 2:**
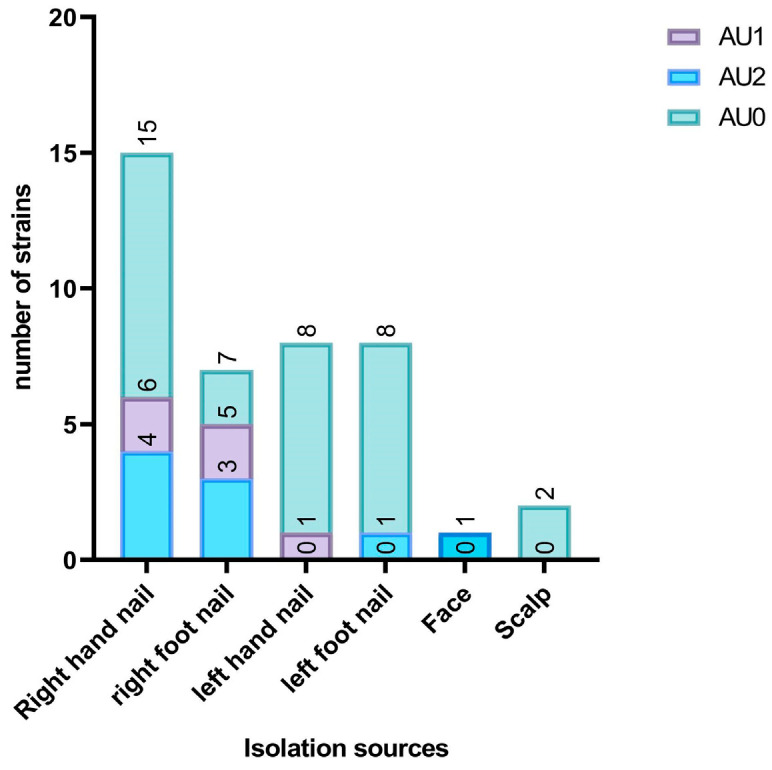
The distribution of arbitrary units by isolation sources of *Candida* spp. strains. Legend: AU1 = 1 arbitrary unit, AU2 = 2 arbitrary units, and AU0 = 0 arbitrary units.

The qualitative analysis of AgNPs showed that most of the *Candida* spp. strains analyzed in this study were identified as having arbitrary unit values of 0 (AU0; 66.12%), 1 (AU1; 19.35%), and 2 (AU2; 14.51%). In correlation with the isolation sources, it was observed that most strains of *Candida* spp. that presented arbitrary units (AU) = 2 and AU = 1 originated from the right hand nail (four and six strains, respectively), followed by the right foot nail (three and five strains, respectively). However, AU = 0 was the predominant identified value for the qualitative screening of AgNPs (see Figure 2).

However, only strains that showed 2 arbitrary units were selected, as these were the most sensitive to the AgNPs tested.

**Figure 3:**
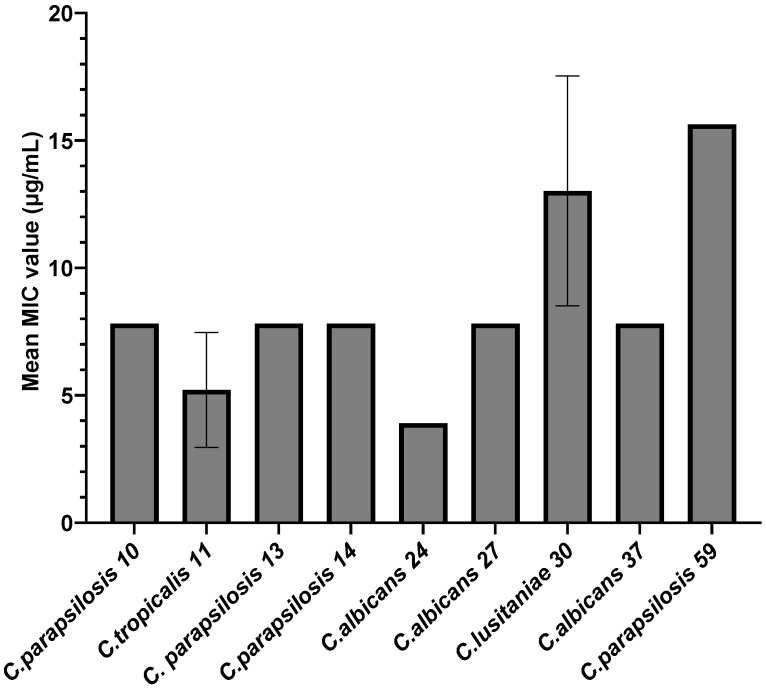
Average MIC values for *Candida* spp. strains.

In the case of the strains for which, following the qualitative testing, an inhibition zone equivalent to 2 arbitrary units was obtained (n = 9), the minimum inhibitory concentration values were also determined. According to Figure 3, the inhibitory effect of AgNPs depends on the species to which the tested strain belongs. Thus, the average MIC values obtained ranged from 3.90 µg/mL to 15.62 µg/mL, with the most sensitive strain being *C. albicans* 24 (MIC = 3.90 µg/mL), followed by *C. tropicalis* 11 (MIC = 5.20 µg/mL), while the most resistant strains were *C. parapsilosis* 59 (MIC = 15.62 µg/mL) and *C. lusitaniae* 30 (MIC = 13.02 µg/mL). For subsequent tests, MIC/2 and MIC/4 were considered, with fractions of the MIC value being determined for each strain.

#### 3.2.2. Anti-Biofilm Activity of AgNPs

**Figure 4:**
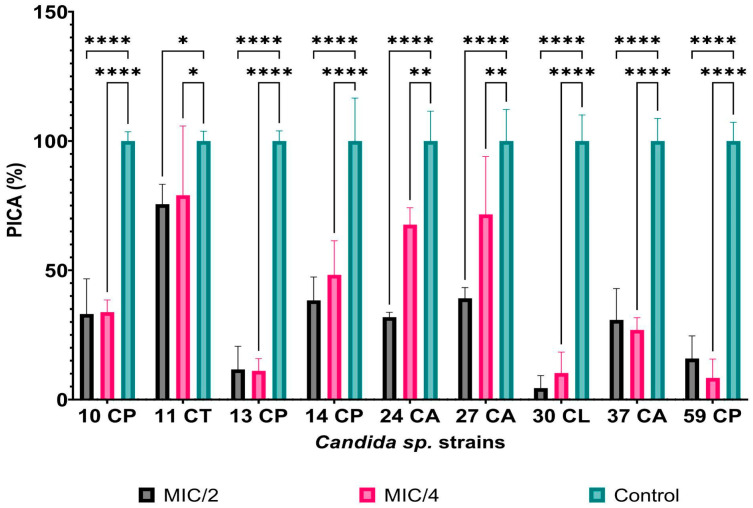
Adherence inhibition percentage (PICA%) values for AgNPs compared to the *Candida* spp. strains (* *p* < 0.05, ** *p* < 0.001, **** *p* < 0.0001) (Dunnett’s multiple comparisons test).

The selected *Candida* strains also presented the ability to develop biofilms on the surface of inert substrata. Thus, we also tested the impact of sub-inhibitory concentrations of AgNPs on their ability to adhere to polypropylene surfaces. According to Figure 4, their adherence ability was significantly reduced at both MIC/2 and MIC/4, the best results being recorded for the *C. lusitaniae* 30 strain, where a reduction of up to 95% in adhesion capacity was observed when it was exposed to MIC/2 AgNPs (PICA% MIC/2 = 4.41%; PICA% MIC/4 = 10.57%). Similar results were obtained for the *C. parapsilosis* 13 (PICA% MIC/2 = 11.66%; PICA% MIC/4 = 11.09%) and *C. parapsilosis* 59 strains (PICA% MIC/2 = 15.89%; PICA% MIC/4 = 8.36%). For the other *C. parapsilosis* strains, the effect was diminished, but the PICA% values remained below 50% when exposed to MIC/2 AgNP concentrations, indicating high efficiency in preventing biofilm formation. Although *C. tropicalis* 11’s growth was strongly inhibited by the presence of AgNPs, the effect of AgNPs on its ability to adhere was limited (PICA% values of up to 75% for MIC/2 and MIC/4). This indicates that, in the case of this strain, the presence of AgNPs induces a generalized state of stress, against which special metabolic pathways are activated, including those associated with adherence. Similarly, for the two *C. albicans* strains, AgNPs reduced their ability to adhere, with the PICA% being 30.814% and 31.89%, respectively, when exposed to MIC/2. 

Although the crystal violet (CV) test serves as an indicator of the biomass attached to a biofilm without revealing the metabolic state of the cells, the MTT test, a tetrazole salt, highlights the presence of metabolic active cells, thus serving as an indicator of the breathing of living cells [58,59]. In biofilms, the MTT test can be used as an indicator of the viable cells attached, while CV colors both viable and nonviable cells that are attached to the inert substrate [60]. The results of the MTT test confirmed that AgNPs significantly inhibited the metabolic activity of biofilms formed by all *Candida* species in this work (*p* < 0.0001) at MIC/2 and MIC/4. The addition of AgNPs prevented the initial attachment of cells, reducing not only the biomass (as indicated by the CV test) but also the metabolic activity of the cells, which resulted in 0.36 ± 0.07% PICV for *C. albicans* strain 37 and 0.10 ± 0.0% PICV for *C. albicans* strain 27 (Table 4). For all strains, the metabolic activity was inhibited as the AgNP concentration increased.

#### 3.2.3. The Impact of AgNPs on Metabolic Activity in Selected *Candida* spp. Isolates

**Figure 5:**
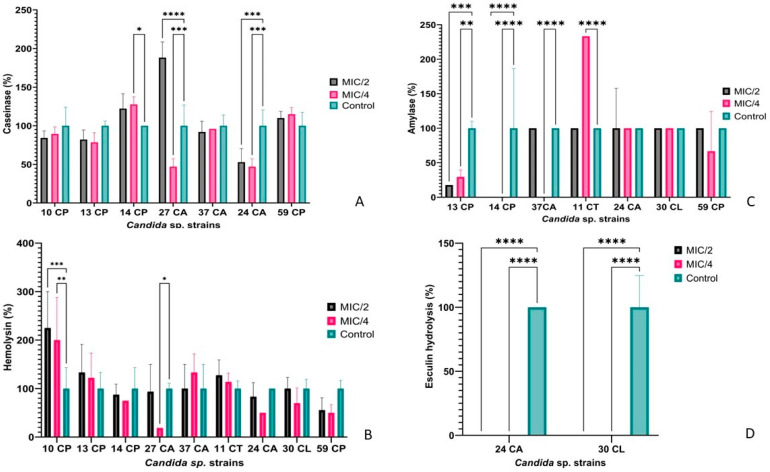
AgNPs’ effects on *Candida* spp. strains’ virulence factors (* *p* < 0.05, ** *p* < 0.001, *** *p* < 0.001, **** *p* < 0.0001) (Dunnett’s multiple comparisons test). (**A**) Caseinase production of *Candida* sp. strains, (**B**) Hemolysis production of *Candida* sp. strains, (**C**) Amylase production of *Candida* sp. strains, (**D**) Esculin Hydrolysis production of *Candida* sp. strains.

Of the tested *Candida* strains, 79.03% were able to hydrolyze casein, and 93.55% hydrolyzed aesculin. Smaller percentages of strains were able to secrete amylase (35.48%), hemolysins (30.65%), and lipases (20.97%). No *Candida* strain was able to produce gelatinases, DNase, or lecithinase. Under these conditions, the strains that presented the capacity to secrete the most varied spectrum of soluble virulence factors and the greatest sensitivity to the antimicrobial action of AgNPs were tested in order to establish the effects of AgNPs on the release of hydrolytic enzymes linked to virulence and pathogenicity. For caseinase production, the best results were obtained in the *C. albicans* 24 strain, for which the secretion capacity was reduced by up to 50% compared to the untreated strain. A similar result was obtained for the *C. albicans* 27 strain treated with MIC/4 AgNPs (47.05%), but when the concentration of AgNPs was increased the effect was the opposite, stimulating the secretion of caseinase in the culture medium (Figure 5A). 

*C. albicans* 24 and *C. lusitaniae* 30 were also tested in the presence of sub-inhibitory concentrations of AgNPs for their ability to hydrolyze aesculin and, according to Figure 5D, their ability was completely inhibited at both MIC/2 and MIC/4 concentrations. 

Amylase production was completely inhibited for the *C. parapsilosis* 14 and *C. albicans* 37 strains when treated with MIC/4 AgNPs. Good results were also obtained for the *C. parapsilosis* 13 strain, the production being reduced by almost 80% in the presence of MIC/2. On the other hand, in the case of *C. tropicalis* 11, it seems that the presence of sub-inhibitory concentrations of AgNPs had a stimulatory effect (Figure 5C). When tested for esterase production, its ability to hydrolyze Tween 80 was completely inhibited in the presence of MIC/4 AgNPs.

Although nine *Candida* strains were tested for hemolysin production in the presence of sub-inhibitory concentrations of AgNPs, only in the case of *C. parapsilosis* 10 was a significant stimulatory effect observed (Figure 5B). 

#### 3.2.4. Extracellular Nitric Oxide Content

**Figure 6:**
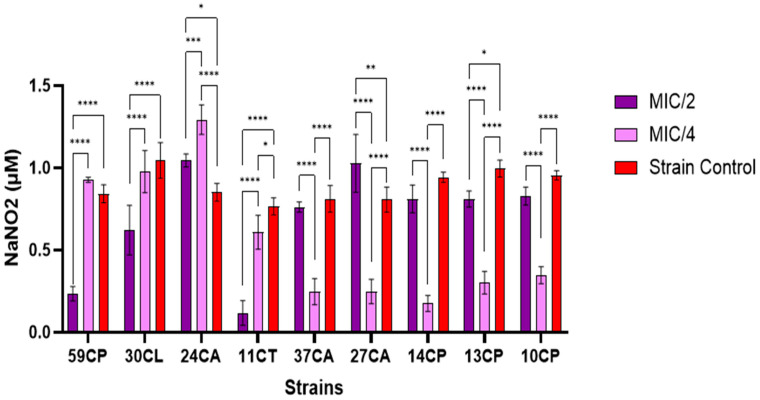
Extracellular NO content determined by the Griess reaction for AgNPs in the presence of *C. albicans* strains (Tukey’s method, * *p* < 0.05, ** *p* < 0.01, *** *p* < 0.001, **** *p* < 0.0001).

**Figure 7:**
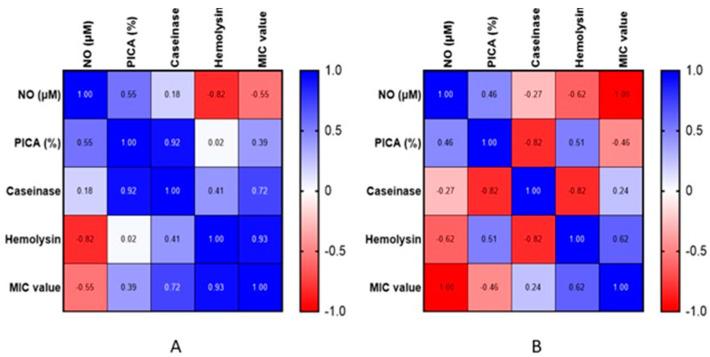
Pearson correlation among extracellular NO content, adhesion inhibition percentage (PICA%), caseinase activity (%), amylase activity (%), and hemolysin (%) for *C. albicans* (**A**) and *C. parapsilosis* (**B**).

Exogenous NO at sublethal doses may induce microorganisms within biofilms to switch from sessile behavior to free-swimming planktonic behavior. NO, at non-lethal levels, has also been shown to increase the susceptibility of various biofilms to antimicrobial treatments [61]. From Figure 6, it can be observed that the concentration of extracellular NO increased with the increase in the AgNPs’ concentration in most *C. parapsilosis* strains, except for 59 CP, which exhibited a higher MIC value compared to other strains. In the case of *C. albicans* strains, it was highlighted that lower sample concentrations generated higher extracellular NO. Among the strains tested at MIC/2, it can be seen that only strains 27CA (*p* < 0.01) and 24CA (*p* < 0.05) showed a significantly higher extracellular NO concentration than the strain control (Figure 7). A significant decrease in extracellular NO concentration was observed compared to the strain control at MIC/4 values for variants 37CA, 27CP, 14CP, 13CP, and 10CP (*p* < 0.0001), which may indicate that the microorganisms used a variety of enzymes and complex processes to recognize, collect, and convert NO into less reactive molecules. Due to its high reactivity, NO can be at inappropriate concentrations and is thus rapidly transformed by microorganisms through the process of aerobic denitrification and oxidation [62].

The anti-biofilm activity of exogenous NO released by low-molecular-weight donors (e.g., AgNP) is dose-dependent; eradication concentrations also depend on the microbial species and potential genetic modifications [55]. The relationship between extracellular NO concentration produced at MIC/2 levels and microbial adhesion revealed that the higher the NO content, the stronger the microbial inhibition of adherence. The Pearson correlation showed that one variable increases as the other grows (r > 0), the correlations being better for the strains of *C. albicans* (r = 0.56) than for *C. parapsilosis* (r = 0.46) (Figure 7A,B). In the case of *C. albicans*, it can be observed that the correlations between NO vs. hemolytic activity (r = −0.82) and NO vs. MIC value (r = −0.55) are inversely proportional, i.e., the higher the extracellular NO content, the lower the hemolysis and MIC value, while the correlations between inhibition of adhesion vs. caseinase activity (r = 0.92), MIC values vs. caseinase activity (r = 0.72), and MIC values vs. hemolytic activity (r = 0.93) are directly proportional, which suggests that the greater the concentration of AgNPs, the higher the caseinase activity and hemolysis, and the better the inhibition of microbial adherence.

In the case of *C. parapsilosis* (Figure 7B), the following correlations can be highlighted: PICA vs. caseinase activity (r = −0.82), PICA vs. hemolysis (r = 0.51), caseinase activity vs. hemolysis (r = 0.82), NO vs. hemolysis (r = −0.62), and sample concentration vs. hemolysis (r = 0.62).

## 4. Discussion

Our obtained results highlighted a high diversity of species responsible for dermatomycoses in the Bucharest community, as follows, in decreasing frequency order: *C. parapsilosis*, *C. albicans*, *C. guilliermondii*, *C. pararugosa*, *C. tropicalis*, *C. lusitaniae*, *C. haemulonii*, *C. famata*, *C. metapsilosis*, and *C. kefyr*. Depending on the identified species, strains exhibited different resistance profiles, e.g., *C. albicans*, *C. parapsilosis*, *C. guilliermondii*, and *C. lusitaniae* were resistant to nystatin and amphotericin B, while *C. albicans*, *C. parapsilosis*, *C. tropicalis*, and *C. haemulonii* were resistant to clotrimazole and econazole. Genotypic assays for the identification of the virulence markers responsible for adherence (*ALS1*, *ALS3*, and *HWP1*) and lipolytic activity (LIP1 and LIP4) showed that most of the tested strains belonging to *C. albicans* were positive for *ALS1* and *HWP1* genes, and *LIP1* in the case of *C. tropicalis*.

The studied strains showed varying ability to form biofilms, differentiated by species as follows: *C. parapsilosis* was categorized as a strong, weak, and moderate biofilm producer in decreasing frequencies of 38.88%, 33.33%, and 5.55%, respectively; *C. albicans* was divided into weak and strong biofilm producers (35.71%/32.14%, respectively); *C. guilliermondii* was predominantly a weak biofilm producer (50%); *C. lusitaniae* had strong biofilm-producing strains (100%); *C. tropicalis* had an equal distribution between weak and strong biofilm formation (50%/50%); and *C. krusei* and *C. parrarugosa* were primarily weak biofilm producers (100% and 50%, respectively), excluding those strains that were not biofilm producers. Our results suggest that the most sensitive strains to AgNPs were *C. albicans* 24 and *C. tropicalis* 11, and at the opposite side were *C. parapsilosis* 59 and *C. lusitaniae* 30. Due to the weak-to-strong biofilm formation capacity of the investigated *Candida* strains, the impact of sub-inhibitory concentrations of AgNPs on their ability to adhere to inert substrata was further investigated, and it was found that the adherence capacity for the most resistant strains was significantly reduced at both MIC/2 and MIC/4 values (e.g., for *C. lusitaniae* 30, a reduction of up to 95% in the adherence capacity was observed after the treatment with MIC/2 AgNPs, followed by *C. parapsilosis* 13 and *C. parapsilosis* 59). 

Strains were positive for aesculin hydrolysis, caseinase, amylase, hemolysins, and lipase production and showed different biofilm formation capacities. Furthermore, results for the adherence capacity of *Candida* spp. strains to eukaryotic cells showed that a high diffuse adherence frequency was obtained. The influence of AgNPs on the secretion of hydrolytic enzymes responsible for virulence and pathogenicity demonstrated that the treatment of microbial cultures with sub-inhibitory concentrations of AgNPs caused stimulatory or inhibitory effects that varied depending on the species, the tested enzyme, or the substrate. 

Previously, Christofidou et al., in a comparative study including Romanian and Greek *Candida* spp. isolates, showed that cutaneous candidiasis is caused mostly by *C. albicans*, followed by *C. parapsilosis*, *Candida glabrata*, *C. tropicalis*, *C. krusei*, *C. guilliermondii*, and other *Candida* spp. resistant to ketoconazole and itraconazole [63]. As in our study, which included *Candida* spp. strains recovered from mucocutaneous candidiasis, several other authors have shown a high prevalence of *NAC* in different clinical sources as a consequence of immunocompromised status, prolonged antifungal therapy, or other administered therapies [64,65,66,67]. In Romania, Sadik et al. (2019) [50], Najee et al. (2018) [68], and Sadik et al. (2017) [69] showed a wide array of adherence genes in Romanian *C. albicans* with varying ability to form biofilms on inert substrata and adherence patterns to eukaryotic cells, with strains isolated from different sources in hospitalized patients aged 20 to 85 and 25 to 89 years [50,68,69].

Several other studies have also highlighted the hemolysin production, adherence markers, and biofilm capacity of *C. albicans* and *NAC*, especially from intra-hospital infection settings, e.g., Singh et al. (2020) [70]. In 2019, Sadeghi et al. showed that *C. albicans* strains prevalent in cases of cutaneous candidiasis in outpatient settings in Tehran, Iran, indicated a change in the disease’s epidemiological patterns, contrasting with our findings. Their research also identified the presence of other *NAC*, e.g., *C. parapsilosis*, *C. tropicalis*, and *C. guilliermondii*—species that were also detected in our study, which are capable of biofilm formation and exhibit phospholipase activity [71]. Mello et al., in 2020, reported the prevalence of *C. albicans*, followed by *C. parapsilosis* complex and *C. krusei* biofilm-producing strains—protease and phospholipase producers isolated from dermatomycosis in Sao Paulo, Brazil, during 2016 and 2017, underscoring the importance of recognizing different fungal species involved in dermatomycosis cases, as well as understanding their pathogenicity and susceptibility to antifungals for effective preventive and therapeutic actions against them [72].

In addition to the classic solutions for treating cutaneous candidiasis based on the use of traditional antifungals (fluconazole, miconazole, clotrimazole, econazole, nystatin, micafungin, anidulafungin, and caspofungin) or topical medications based on potassium permanganate, gentian violet solution, or aluminum acetate solution [73], AgNPs could be a promising tool for treating dermatomycosis, due to their known antifungal activity via different mechanisms, including ion release, oxidative and nitrosative stress, damage to membranes and cell walls, inhibition of biochemical processes, reduction in ATP levels, and dysfunction in DNA, proteins, and mitochondria [63,64], as supported by several studies [74,75]. AgNPs’ effects on human cells are complex and depend on several factors, including their size, coating, concentration, and the specific types of human cells they interact with. In terms of size, smaller NPs tend to have a higher cytotoxic effect due to their greater surface area–volume ratio, which facilitates more interactions with cellular components. However, certain coatings can mitigate these effects by providing a barrier that reduces cellular uptake and toxicity [76] Concerning human cell types, different cells exhibit varying levels of sensitivity to AgNPs. For instance, human hepatoma cells (HepG2) and human lung epithelial cells have shown different responses to AgNP exposure, with some studies indicating that specific coatings or modifications can reduce cytotoxicity while maintaining antimicrobial efficacy [77,78]. AgNPs can induce apoptosis and necrosis in human osteoblast cells through oxidative damage and nitric oxide signaling pathways [77,78].

Despite their potential cytotoxic effects, AgNPs are being explored for their therapeutic benefits, such as antiviral and antibacterial properties. Studies have demonstrated that AgNPs can inhibit the replication of viruses and bacteria at non-cytotoxic concentrations, suggesting their potential use in medical applications without significant harm to human cells [76].

Comparable to our findings, high antifungal activity of AgNPs has been demonstrated in *C. albicans* and *NAC* isolates recovered from different isolation sources—including skin swabs, nails, oral swabs, cervical swabs, urine, sputum, endotracheal aspirates—that are resistant to fluconazole, ketoconazole, clotrimazole, itraconazole, amphotericin B, and nystatin [79]. The anti-biofilm properties of biogenic AgNPs against reference strains of *C. albicans*, as well as the inhibition of yeast-to-hyphal transition, increased the membrane permeabilization, ROS production, and oxidative stress, and finally led to apoptosis [80]. Also, the influence of AgNPs against *C. albicans* biofilms was explored, emphasizing the pressing demand for new antifungal treatments due to the biofilms’ resistance to conventional antifungal agents. This study revealed that AgNPs exhibit potent inhibitory effects on biofilm formation and against pre-formed biofilms of *C. albicans*. Furthermore, our findings underscore the potential of AgNPs as a promising treatment strategy against *C. albicans* infections, demonstrating that AgNPs target both growth stages by disrupting the cell membrane structure of the fungal cells. AgNPs show significant antifungal activity, suggesting their role as instrumental in combating biofilm-associated candidiasis, offering a new avenue for antifungal intervention [39]. Several other studies have demonstrated the antimicrobial activity of AgNPs against *C. albicans*, *C. dubliniensis*, and *C. guilliermondii* through qualitative and quantitative assays, as well as the capacity of uninvestigated NPs to disrupt the cell membrane/wall based on microscopic examination [81]. Furthermore, Vazquez-Muñoz demonstrated the fungicidal effect of AgNPs and their ability to reduce cell viability when combined with fluconazole. Ultrastructural analysis revealed the presence of AgNPs both outside and inside the cells, suggesting a potential mechanism of action. Their study also discussed the potential clinical implications of using AgNPs in combination with antifungals and highlighted the need for further research to understand their safety and effectiveness [82].

The effectiveness of AgNPs in combating fungal infections in plants and humans was also demonstrated, with a specific emphasis on *Candida* spp., highlighting that AgNPs effectively inhibit the growth of *Candida* spp. and influence the production of virulence factors, e.g., biofilm formation. Furthermore, research supports the notion that AgNPs not only reduce yeast cell proliferation but also impact various virulence determinants, including biofilm maturation [83]. In 2022, the fungicidal properties of AgNPs were demonstrated against resistant strains of *C. albicans* and *NAC*, isolated from both outpatients and inpatients in Egypt. The authors proposed that AgNPs could serve as a viable alternative to traditional antifungal agents [84]. Moreover, the increased hemolysin production in the presence of AgNPs described in this study was observed previously, enhancing the pathogenicity of these fungi, possibly due to interactions with fungal cell membranes or metabolic processes [85]. Comparative research shows that both *C. albicans* and *NAC* strains respond similarly to NP exposure [86]. Further studies suggest that NPs not only compromise the structural integrity of *Candida* biofilms but also stimulate hemolysin production, impacting the biofilm’s resilience and offensive capabilities [39,87]. These findings point to a potential for using silver nanoparticles to modulate fungal virulence, although more research is needed to explore the mechanisms and therapeutic possibilities. 

Furthermore, AgNPs act as carrier systems for different antifungal agents, e.g., the synergistic interaction of AgNPs and fluconazole was demonstrated in *C. albicans* planktonic cells [88], with a decrease in ergosterol levels and cell membrane disruption by downregulating the *ERG1*, *ERG11*, and *ERG25* genes, and cellular damage as a consequence of the ROS production [89]. Several other studies have highlighted the synergistic interactions of AgNPs functionalized with poly(methacrylic acid) and fluconazole in fluconazole-resistant *C. albicans* [90], as well as the synergistic effects of biogenic AgNPs derived from *Anabaena variabilis* and fluconazole against *C. albicans* planktonic cells [80], or the antifungal effects and the impact on biochemical processes of green-synthesized silver–copper nanoparticles against *Candida* strains [91].

This study’s findings might be limited by its small sample size and specific demographic focus, having analyzed only 62 *Candida* spp. strains previously isolated from Bucharest outpatients (2021). This narrow geographic and temporal scope may not capture the full diversity and potential variations in antimicrobial resistance and virulence traits of *Candida* strains found in other regions or over different periods. Such limitations could hinder the broader applicability of the results to other settings or over time, as treatment practices and environmental factors evolve. 

The use of qualitative (adapted disk diffusion method) and quantitative (serial twofold microdilutions) assessments might not fully capture the dynamics of nanoparticle–fungal interactions, particularly if the nanoparticles form aggregates or interact differentially with the assay components. Additionally, this study’s reliance on in vitro assays to predict clinical efficacy could be a limitation, as these conditions do not fully replicate the complex environment of a human infection, including immune responses and the presence of other microbiota.

These limitations suggest that, while this study provides valuable insights into the potential of AgNPs against *Candida* infections, the results should be interpreted with caution, considering the need for further characterization of the nanoparticles and validation of the findings in more clinically relevant models.

Considering the limited number of studies that have examined the relationship between biofilm production by dermatomycosis isolates and their consequences on pathogenesis of infections, our study concentrated on the phenotypic and genotypic traits of 62 *Candida* spp. previously isolated from community-acquired infections in the Central Reference Synevo-Medicover Laboratory, Bucharest, Romania, as well as on the efficiency of alternative solutions based on AgNPs to limit the spread of virulent *Candida* spp. strains. This study has significant implications for both practice and policy. The findings enhance our understanding of the characteristics and behaviors of these strains, which is crucial for developing targeted treatment strategies. Moreover, this study’s exploration of alternative solutions based on AgNPs presents a promising tool for limiting the spread of virulent *Candida* strains. This approach could potentially improve treatment outcomes and reduce the reliance on traditional antifungal agents, which often face resistance issues. Therefore, this study supports the need for integrating advanced diagnostic methods and innovative treatments into clinical practice and informing policy decisions aimed at controlling *Candida* infections more effectively. Future research should focus on the following: understanding how these *Candida* spp. develop resistance to conventional antifungal agents, as well as how AgNPs can overcome these mechanisms; evaluating the safety and efficacy of AgNPs in treating *Candida* infections, ensuring that they are a viable alternative to current therapies; exploring the most effective ways to administer AgNPs, whether through topical, systemic, or combined therapies; investigating the long-term impacts of AgNP use on both patients and microbial communities, to ensure that there are no adverse effects; comparing the effectiveness of AgNPs with other emerging antifungal agents; and providing data to support the development of guidelines and policies for the clinical use of AgNPs, ensuring that they are appropriately integrated into treatment protocols. Overall, this study highlights the need for continued research into alternative solutions based on AgNPs to address the growing challenge of antifungal resistance in *Candida* spp.

## 5. Conclusions

Considering the scarcity of research on the virulence of *Candida* spp. in Romanian dermatomycosis cases, this study employed a multifaceted approach. 

This study represents the first characterization of *Candida* spp. strains isolated from dermatomycosis in ambulatory patients from Bucharest, the capital city of Romania, and provides valuable insights into the prevalence, diversity, and antifungal susceptibility of these pathogens. This study found that a variety of *Candida* species are responsible for dermatomycosis in this urban population, highlighting the importance of accurate identification for effective treatment. Notably, our findings indicate a concerning trend of antifungal resistance among certain strains, underscoring the need for ongoing surveillance and tailored therapeutic strategies. This initial characterization serves as a crucial step towards understanding the epidemiology of *Candida* infections in Bucharest, ultimately contributing to better clinical management and control measures in the region. Furthermore, the obtained results constitute a preliminary study suggesting the potential use of AgNPs as therapeutic alternatives to combat *Candida* spp. strains causing dermatomycosis.

## Figures and Tables

**Table 1:** Phenotypic characterization of *Candida* spp. ambulatory strains.

Species/Strain Number	Isolation Sources	Patient Ages	Sex	Resistance Profiles
*C. albicans* (*n* = 18)	Left and right foot, right and left hand nail, face scales	26–73 years	Male and female	Amphotericin B (CMI > 2 µg/mL) (22%), clotrimazole (CMI > 1 µg/mL) (27%), nystatin (CMI > 1.25 µg/mL) (17%), econazole (CMI > 2 µg/mL) (11%), miconazole (CMI > 2 µg/mL) (6%),
*C. parapsilosis* (*n* = 28)	Left and right foot, right and left hand nail, scalp	25–77 years	Male and female	Amphotericin B (CMI > 2 µg/mL) (29%), clotrimazole (CMI > 1 µg/mL) (24%), nystatin (CMI > 1.25 µg/mL) (18%), econazole (CMI > 2 µg/mL) (11%), miconazole (CMI > 2 µg/mL) (7%), itraconazole (CMI > 1 µg/mL) (4%),
*C. tropicalis* (*n* = 2)	Left foot	31–72 years	Male and female	Clotrimazole (CMI > 1 µg/mL), econazole (CMI > 2 µg/mL) and nystatin (CMI > 1.25 µg/mL) (50/50/%)
*C. guilliermondii* (*n* = 6)	Left foot, right and left hand nail, scalp	42–75 years	Male and female	Nystatin (17%) (CMI > 1.25 µg/mL)
*C. lusitaniae* (*n* = 2)	Right hand nail	42 to 54 years	Female	Nystatin (CMI > 1.25 µg/mL) and amphotericin B (CMI > 2 µg/mL) (100%)
*C. krusei* (*n* = 1)	Left hand nail	55 years	Female	-
*C. metapsilosis* (*n* = 1)	Right hand nail	31 years	Female	-
*C. haemulonii* (*n* = 1)	Left hand nail	47 years	Female	Clotrimazole (CMI > 1 µg/mL) and econazole (CMI > 2 µg/mL) (100%)
*C. famata* (*n* = 1)	Left foot	45 years	Female	-
*C. pararugosa* (*n* = 2)	Left and right hand nail	15–28 years	Female	-

“-“ states that no resistance profile was determined.

**Table 2:** Phenotypic and genotypic virulence markers of *Candida* spp. strains.

Species/Strain Number	Virulence Factors/Metabolic Enzymes	Genotypic Traits
		VMs, Adherence, Lipolytic Activity
*C. albicans (n* = 18)	Aesculin hydrolysis, hemolysins, amylase, lipase	*ALS1*, *ALS3*, *HWP1*
*C. parapsilosis* (*n* = 28)	Aesculin hydrolysis, caseinase hemolysins, amylase, lipase	N/A
*C. tropicalis* (*n* = 2)	Aesculin hydrolysis, caseinase hemolysins, amylase	*LIP 1*
*C. guilliermondii* (*n* = 6)	Aesculin hydrolysis, caseinase hemolysins, amylase, lipase	N/A
*C. lusitaniae* (*n* = 2)	Aesculin hydrolysis, caseinase hemolysins, amylase, lipase	N/A
*C. krusei* (*n* = 1)	Aesculin hydrolysis, amylase	N/A
*C. metapsilosis* (*n =* 1)	Aesculin hydrolysis, caseinase	N/A
*C. haemulonii* (*n =* 1)	-	N/A
*C. famata* (*n =* 1)	Caseinase	N/A
*C. pararugosa* (*n =* 2)	Aesculin hydrolysis	N/A

“N/A”—not assessed, “-“ states that no enzyme was detected.

**Table 3:** Biofilm and adherence capacity of *Candida* spp. strains.

Species/Strain Number	Biofilm Formation Capacity on Inert Substratum	Adherence Pattern to HeLa Cells	AIn
*C. albicans* (*n* = 18)	NP(4)	Diffuse adherence	39.5%
	W (6)		
	M (1)		
	S (7)		
*C. parapsilosis* (*n* = 28)	NP (9)	Localized adherence	50%
	W (10)		
	S (9)		
*C. tropicalis* (*n* = 2)	W(1)	Aggregative adherence	35%
	S (1)		
*C. guilliermondii* (*n* = 6)	NP (3)	Localized adherence	28.5%
	W (3)		
*C. lusitaniae* (*n* = 2)	S (2)	Not tested	Not tested
*C. krusei* (*n* = 1)	W (1)	Localized adherence	26%
*C. metapsilosis* (*n =* 1)	NP (1)	Not tested	Not tested
*C. haemulonii* (*n =* 1)	NP (1)	Not tested	Not tested
*C. famata* (*n =* 1)	NP (1)	Not tested	Not tested
*C. pararugosa* (*n =* 2)	NP(1)	Not tested	Not tested
	W(1)		

NP—biofilm non-producers; W—weak, M—moderate, S—strong biofilm-producing strains AIn—adherence index; VMs—virulence markers.

**Table 4:** The AgNPs’ MIC values and the corresponding MIC/2 and MIC/4 for PICV (%) determination in selected *Candida* spp. strains.

Strain	MIC/2 (μg/mL)	PICV%	*p*-Value	MIC/4 (μg/mL)	PICV%	*p*-Value
10 *C. parapsilosis*	3.90	38.98 ± 0.51%	<0.0001	1.95	45.88 ± 1.37%	<0.0001
11 *C. tropicalis*	2.60	18.72 ± 0.88%	<0.0001	1.30	75.26 ± 2.15%	<0.0001
13 *C. parapsilosis*	3.90	44.95 ± 5.67%	<0.0001	3.90	65.23 ± 2.49%	<0.0001
14 *C. parapsilosis*	3.90	6.43 ± 0.23%	<0.0001	1.95	54.80 ± 1.51%	<0.0001
24 *C. albicans*	1.95	10.84 ± 0.34%	<0.0001	0.97	13.95 ± 0.28%	<0.0001
27 *C. albicans*	3.90	0.10 ± 0.02%	<0.0001	1.95	46.82 ± 2.50%	<0.0001
30 *C. lusitaniae*	6.51	0.39 ± 0.03%	<0.0001	3.25	66.69 ± 8.88%	<0.0001
37 *C. albicans*	3.90	0.36 ± 0.07%	<0.0001	1.95	2.98 ± 0.07%	<0.0001
59 *C. parapsilosis*	7.81	22.53 ± 2.30%	<0.0001	3.90	43.02 ± 0.81%	<0.0001

## Data Availability

AgNP samples and previous characterization, *Candida* spp. isolates, and datasets pertaining to the results can be obtained from the authors.

## Supplementary Materials

The following supporting information can be downloaded at: https://www.mdpi.com/article/10.3390/jof10080563/s1, Table S1: *Candida albicans* and *Candida* non-*albicans* strains investigated in the study; Table S2: Primers for virulence genes detection; Table S3: Amplification programs for virulence genes detection; Table S4: The AgNPs’ MIC values and the corresponding MIC/2 and MIC/4 for PICA determination in selected *Candida* spp. strains; Table S5: The influence of AgNPs sub-inhibitory concentrations (MIC/2) on the virulence factor production in selected *Candida* spp. strains; Table S6: The influence of AgNPs sub-inhibitory concentrations (MIC/4) on the virulence factor production in selected *Candida* spp. strains; Figure S1: Electrophoresis gel for *LIP1* gene (1900 bp) gene detection; Figure S2: Electrophoresis gel for *LIP4* gene (2300 bp) gene detection; Figure S3: Electrophoresis gel for *HWP1* gene (570 bp) gene detection; Figure S4: Electrophoresis gel for *ALS1* gene (320 bp) gene detection; Figure S5: Electrophoresis gel for *ALS3* gene (185 bp) gene detection.
