# Supplementary material for: Phenotypic and Genotypic Characterization of Resistance and Virulence Markers in Candida spp. Isolated from Community-Acquired Infections in Bucharest, and the Impact of AgNPs on the Highly Resistant Isolates
Source: J Fungi (Basel). 2024 Aug 9;10(8):563. doi: 10.3390/jof10080563 (PMC11355189; doi:10.3390/jof10080563)
Supplement: Supplementary file 1 [file jof-10-00563-s001.zip › jof-3133036-supplementary.pdf]

Journal of *Fungi*

MDPI

Supplementary Material

Phenotypic and genotypic characterization of resistance and virulence markers in *Candida* spp. isolated from the community acquired infections in Bucharest

Viorica Maria Corbu[1,2]†, Ana-Maria Georgescu*[1]†, Ioana Cristina Marinas†[2], Radu Pericleanu[1], Denisa Vasilica Mogos[1], Andreea Ștefania Dumbravă[1,2], Liliana Marinescu[4], Ionut Pecete[3], Tatiana Vassu Dimov[1], Ilda Czobor Barbu[1,2], Ortansa Csutak[1,2], Denisa Ficai[4,5], Irina Gheorghe-Barbu[1,2]†

1. Supplementary Figures and Tables

1.1. Supplementary Tables

Supplementary Table 1. ***Candida albicans*** **and** ***Candida*** **non** ***albicans*** **strains investigated in the study.**

Strain code	Species	Isolation source	Patient sex	Patient age
1 CP	*C. parapsilosis*	Right foot	F	45 years
2 CPa	*C. pararugosa*	Right hand nail	F	50 years
3 CP	*C. parapsilosis*	Right hand nail	F	48 years
4 CA	*C. albicans*	Right foot	F	54 years
5 CA	*C. albicans*	Left foot	F	30 years
6 CA	*C. albicans*	Right foot	M	75 years
7 CA	*C. albicans*	Right hand nail	F	55 years
8 CPa	*C. pararugosa*	Left hand nail	F	40 years
9 CP	*C. parapsilosis*	Right foot	M	72 years
10 CP	*C. parapsilosis*	Left foot	F	55 years
11 CT	*C. tropicalis*	Left foot	F	63 years

12 CM	*C. metapsilosis*	Right hand nail	F	58 years
13 CP	*C. parapsilosis*	Right hand nail	F	54 years
14 CP	*C. parapsilosis*	Left foot	M	77 years
15 CP	*C. parapsilosis*	Right hand nail	F	42 years
16 CA	*C. albicans*	Right hand nail	F	58 years
17 CA	*C. albicans*	Right hand nail	F	43 years
18 CP	*C. parapsilosis*	Right foot	F	56 years
19 CG	*C. guilliermondii*	Left hand nail	M	64 years
20 CG	*C. guilliermondii*	Right hand nail	M	72 years
21 CP	*C. parapsilosis*	Right hand nail	M	73 years
22 CF	*C. famata*	Right foot	F	66 years
23 CP	*C. parapsilosis*	Left foot	M	31 years
24 CA	*C. albicans*	Right hand nail	M	54 years
25 CA	*C. albicans*	Right foot	M	61 years
26 CH	*C. haemulonii*	Left hand nail	F	50 years
27 CA	*C. albicans*	Left foot	F	70 years
28 CA	*C. albicans*	Right foot	F	41 years
29 CA	*C. albicans*	Right hand nail	F	66 years
30 CL	*C. lusitaniae*	Right hand nail	F	47 years
31 CL	*C. lusitaniae*	Right hand nail	F	35 years
32 CP	*C. parapsilosis*	Right foot	M	28 years
33 CA	*C. albicans*	Left foot	M	67 years
34 CA	*C. albicans*	Right hand nail	F	39 years
35 CK	*C. krusei*	Left hand nail	F	63 years
36 CA	*C. albicans*	Right hand nail	F	57 years
37 CA	*C. albicans*	Face	M	67 years
38 CA	*C. albicans*	Right foot	F	68 years

39 CA	*C. albicans*	Right hand nail	F	50 years
40 CP	*C. parapsilosis*	Right hand nail	F	28 years
41 CP	*C. parapsilosis*	Left hand nail	M	58 years
42 CP	*C. parapsilosis*	Left hand nail	F	45 years
43 CP	*C. parapsilosis*	Right hand nail	M	54 years
44 CP	*C. parapsilosis*	Right foot	M	31 years
45 CP	*C. parapsilosis*	Right hand nail	F	63 years
46 CP	*C. parapsilosis*	Left foot	M	69 years
47 CP	*C. parapsilosis*	Right hand nail	F	26 years
48 CP	*C. parapsilosis*	Right foot	M	64 years
49 CG	*C. guilliermondii*	scalp	M	42 years
50 CP	*C. parapsilosis*	Right foot	F	69 years
52 CP	*C. parapsilosis*	face	M	38 years
51 CP	*C. parapsilosis*	Right hand nail	F	15 years
53 CG	*C. guilliermondii*	Left foot	F	65 years
54 CP	*C. parapsilosis*	Right hand nail	F	33 years
55 CG	*C. guilliermondii*	Right foot	M	47 years
56 CP	*C. parapsilosis*	scalp	M	75 years
57 CP	*C. parapsilosis*	Left hand nail	F	25 years
57 CT	*C. tropicalis*	Left foot	M	28 years
58 CA	*C. albicans*	Left hand nail	F	47 years
59 CP	*C. parapsilosis*	Right hand nail	F	57 years
60 CG	*C. guilliermondii*	Right hand nail	M	28 years
61 CP	*C. parapsilosis*	Left hand nail	F	30 years

Supplementary Table 2. Primers for virulence genes detection.

Genes	Primers	Amplicon size	Primer sequences	References
LIP1	LIP1 F	1945 bp	5`-CCA AGG AGT CTA TGG CTC AGT TA-3`	Zhang et al., 2019
	LIP1 R		5`-TAA GTG TAA AGT TGT CGG TGT TC-3`	
LIP4	LIP4 F	2300 bp	5`-AAA CCA GCG ACA CCA ACC TAC AA-3`	Dikmen et al., 2021
	LIP4 R		5`-TGG TGG AAA GAC AGG TCG CAG TT-3`	
ALS1	ALS1 F	320 bp	5`-ACC AGA AGA AAC AGC AGG TG-3`	
	ALS1 R		5`-GAC TAG TGA ACC AAC AAA TAC CAG-3`	
ALS3	ALS3 F	185 bp	5`-CCA AGT GTT CCA ACA ACT GAA-3`	
	ALS3 R		5`-GAA CCG GTT GTT GCT ATG GT-3`	
HWP1	HWP1 F	570 bp	5`-ATG ACT CCA GCT GGT TC-3`	
	HWP1 R		5`-TAG ATC AAG AAT GCA GC-3`	

Supplementary Table 3. Amplification programs for virulence genes detection.

Genes	Amplification program			
	Denaturation	Annealing	Extension	No. of cycles
LIP1	**94°C, 3 min**	**8.3°C, 30 s**	**72°C, 4 min**	**30**
LIP4	**94°C, 3 min**	**51°C, 30 s**	**72°C, 4 min**	**30**

ALS1	**94°C, 4 min**	**50°C, 1 min**	**72°C, 1 min**	**35**
ALS3	**94°C, 4 min**	**51°C, 1 min**	**72°C, 1 min**	**30**
HWP1	**94°C, 4 min**	**59°C, 1 min**	**72°C, 1 min**	**35**

Supplementary Table 4. The AgNPs' MIC values and the corresponding MIC/2 and MIC/4 for PICA determination in selected *Candida* spp. strains.

**Strain**	**Isolation source**	**MIC (µg/mL)**	**MIC/2 (µg/mL)**	**PICA%**	**p-value**	**MIC/4 (µg/mL)**	**PICA%**	**p-value**
10 *C. parapsilosis*	Left foot	7.81	3.90	33.13%	<0.0001	1.95	33.80%	<0.0001
11 *C. tropicalis*	Left foot	5.20	2.60	75.55%	0.0140	1.30	79.06%	0.0383
13 *C. parapsilosis*	Right hand nail	7.81	3.90	11.67%	<0.0001	3.90	11.10%	<0.0001
14 *C. parapsilosis*	Right foot	7.81	3.90	38.36%	<0.0001	1.95	48.26%	<0.0001
24 *C. albicans*	Right hand nail	3.90	1.95	31.89%	<0.0001	0.97	67.62%	0.0011
27 *C.albicans*	Left foot	7.81	3.90	39.17%	<0.0001	1.95	71.62%	0.0041
30 *C.lusitaniae*	Right hand nail	13.02	6.51	4.41%	<0.0001	3.25	10.27%	<0.0001
37 *C. albicans*	Face	7.81	3.90	30.81%	<0.0001	1.95	26.95%	<0.0001
59 *C. parapsilosis*	Left hand nail	15.625	7.81	15.89%	<0.0001	3.90	8.37%	<0.0001

Supplementary Table 5. The influence of AgNPs sub-inhibitory concentrations (MIC/2) on the virulence factor production in selected *Candida* spp. strains

Strain	Isolation source	MIC/2									
		Caseinase		Amylase		Hemolysin		Esterase		Esculin hydrolysis	
		AgNPs	p-value	AgNPs	p-value	AgNPs	p-value	AgNPs	p-value	AgNPs	p-value
10 *C. parapsilosis*	Left foot	84.21053	0.3199	N/A	N/A	225	0.0004	N/A	N/A	N/A	N/A
11 *C. tropicalis*	Left foot	N/A	N/A	100	>0.9999	127.5862	0.5859	94.11765	0.0001	N/A	N/A
13 *C. parapsilosis*	Right hand nail	82.14286	0.2410	17.64706	0.0008	133.3333	0.4666	N/A	N/A	N/A	N/A
14 *C. parapsilosis*	Right foot	122.2222	0.1226	0	<0.0001	87.5	0.8912	N/A	N/A	N/A	N/A
24 *C. albicans*	Right hand nail	52.94118	0.0006	100	>0.9999	83.33333	0.8166	N/A	N/A	0	<0.0001
27 *C.albicans*	Left foot	188.2353	<0.0001	N/A	N/A	93.75	0.9713	N/A	N/A	N/A	N/A
30 *C.lusitaniae*	Right hand nail	N/A	N/A	100	>0.9999	100	>0.9999	N/A	N/A	0	<0.0001
37 *C. albicans*	Face	92	0.7273	100	>0.9999	100	>0.9999	N/A	N/A	N/A	N/A
59 *C. parapsilosis*	Left hand nail	110	0.6137	100	>0.9999	55.55556	0.2743	N/A	N/A	N/A	N/A

Supplementary Table 6. The influence of AgNPs sub-inhibitory concentrations (MIC/4) on the virulence factor production in selected *Candida* spp. strains.

Strain	Isolation source	MIC/4									
		Caseinase		Amylase		Hemolysin		Esterase		Esculin hydrolysis	
		AgNPs	p-value	AgNPs	p-value	AgNPs	p-value	AgNPs	p-value	AgNPs	p-value
10 *C. parapsilosis*	Left foot	89.47368	0.5840	N/A	N/A	200	0.0046	N/A	N/A	N/A	N/A
11 *C. tropicalis*	Left foot	N/A	N/A	233.3333	<0.0001	113.7931	0.8696	0	0.0001	N/A	N/A
13 *C. parapsilosis*	Right hand nail	78.57143	0.1397	29.41176	0.0038	122.2222	0.7020	N/A	N/A	N/A	N/A
14 *C. parapsilosis*	Right foot	127.7778	0.0452	0	<0.0001	75	0.6419	N/A	N/A	N/A	N/A

24 *C. albicans*	Right hand nail	47.05882	0.0001	100	>0.9999	50	0.2020	N/A	N/A	0	<0.0001
27 *C.albicans*	Left foot	47.05882	0.0001	N/A	N/A	18.75	0.0231	N/A	N/A	N/A	N/A
30 *C.lusitaniae*	Right hand nail	N/A	N/A	100	>0.9999	70	0.5347	N/A	N/A	0	<0.0001
37 *C. albicans*	Face	96	0.9212	0	<0.0001	133.3333	0.4666	N/A	N/A	N/A	N/A
59 *C. parapsilosis*	Left hand nail	115	0.3542	66.66667	0.2170	50	0.2020	N/A	N/A	N/A	N/A

1.2. Supplementary Figures

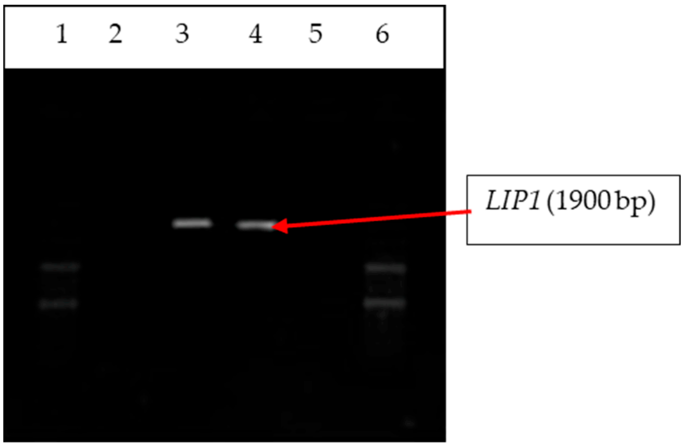

Supplementary Figure 1. Electrophoresis gel for *LIP1* gene (1900 bp) gene detection: all strains of *C. tropicalis* were positives. Lines: **1**- Molecular Size Marker (ThermoScientific)- Ladder Bench Top 100bp; **2**- negative control; **3**- 57 CT; **4**- 11 CT; **5**- negative control; **6**- Molecular Size Marker (ThermoScientific)- Ladder Bench Top 100bp.

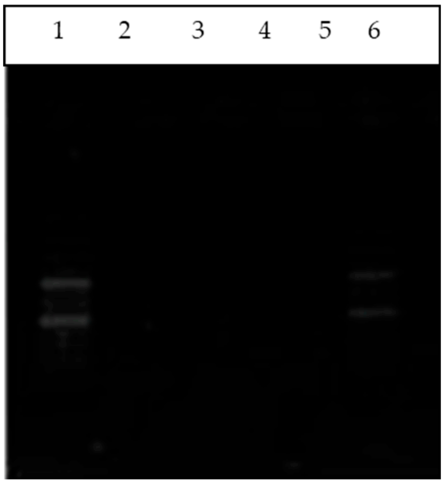

Supplementary Figure 2. Electrophoresis gel for *LIP4* gene (2300 bp) gene detection: all strains of *Candida tropicalis* were negatives. Lines: **1**- Molecular Size Marker (ThermoScientific)- Ladder Bench Top 100bp; **2**- negative control; **3**- 57 CT; **4**- 11 CT; **5**- negative control; **6**- Molecular Size Marker (ThermoScientific)- Ladder Bench Top 100bp.

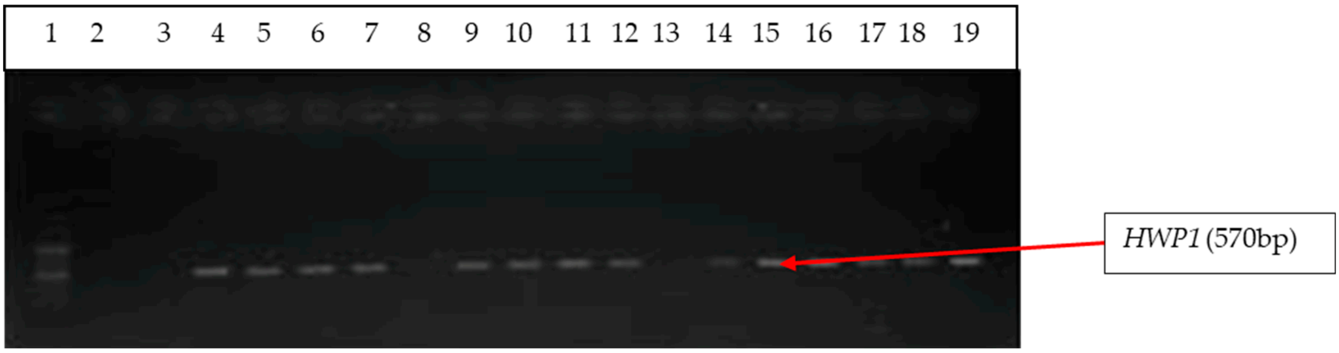

Supplementary Figure 3. Electrophoresis gel for *HWP1* gene (570 bp) gene detection: 14 *Candida* spp. strains were positives. Lines: **1**- Molecular Size Marker (ThermoScientific)- Ladder Bench Top 100bp; **2**- 2 CPr; **3**- negative control;

4-4 CA; **5**-5 CA; **6**-9 CP; **7**-7 CA; **8**-12 CM; **9**-15 CA; **10**-17 CA; **11**-39 CA; **12** –38 CA; 13-24 CA; 14-25 CA; 15-28 CA; 16-29 CA; 17-33 CA; 18- 36 CA; 19- 58 CA.

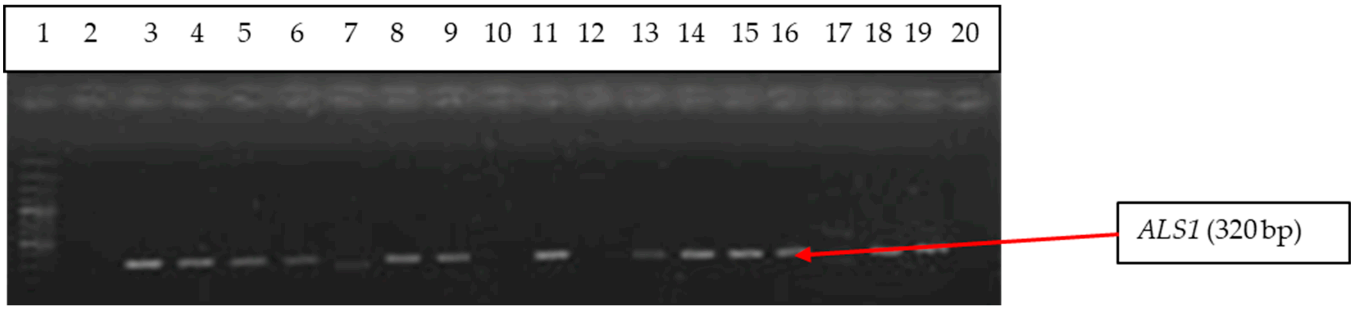

Supplementary Figure 4. Electrophoresis gel for *ALS1* gene (320 bp) gene detection: 14 *Candida* spp. strains were positives. Lines: **1**- Molecular Size Marker (ThermoScientific)- Ladder Bench Top 100bp; **2**- 2 CPr; **3**- 4 CA; **4**-5 CA; **5**-6 CA; **6**-7 CA; **7**-12 CM; **8**-17 CA; **9**-61 CA; **10**- negative control; **11**- 16 CA; **12** – 58 CA; 13- 24 CA; 14-25 CA; 15-28 CA; 16-29 CA; 17-33 CA; 18- 36 CA; 19- 38 CA; 20 – 39 CA.

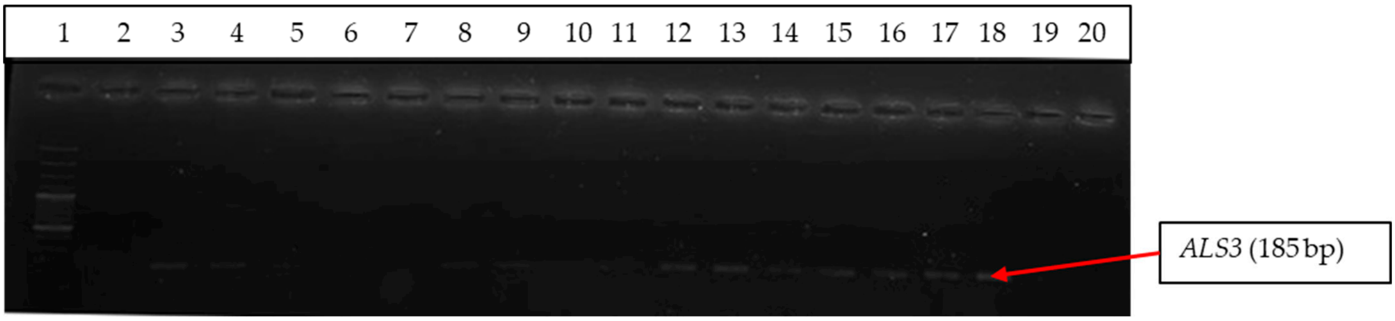

Supplementary Figure 5. Electrophoresis gel for *ALS3* gene (185 bp) gene detection: 10 *Candida* spp. strains were positives. Lines: **1**- Molecular Size Marker (ThermoScientific)- Ladder Bench Top 100bp; **2**- 2 CPr; **3**- 4 CA; **4**-5 CA; **5**-6 CA; **6**-7 CA; **7**-12 CM; **8**-17 CA; **9**-61 CA; **10**- negative control; **11**- 16 CA; **12** – 58 CA; **13**- 24 CA; **14**-25 CA; **15**-28 CA; **16**-29 CA; **17**-33 CA; **18**- 36 CA; **19**- 38 CA; **20** – 39
